# Superior hypogastric nerve block versus spinal anesthesia for early pain control after uterine fibroid embolization: a prospective quasi-randomized comparative study

**DOI:** 10.1186/s42155-026-00749-w

**Published:** 2026-08-27

**Authors:** Priscila Henriques da Silva, Gustavo Borges Teixeira Mendes, Thiago Franchi Nunes, Vinicius Adami Vayego Fornazari, Gloria Maria Martinez Salazar, Andrea Puchnick, Suzan Menasce Goldman, Denis Szejnfeld

**Affiliations:** 1 Department of Diagnostic Imaging, Paulista School of Medicine – Federal University of São Paulo (UNIFESP), Rua Napoleão de Barros, 800, Vila Clementino, São Paulo, SP 04024-002 Brazil; 2 Interventix, Rua Dr. Antônio Alves Arantes, 398, Chácara Cachoeira, Campo Grande, MS 79040-720 Brazil; 3 RAINTER Medical Institute, Av. Ibirapuera, N° 2907 - Conjuntos 901 e 902, São Paulo, SP 04029-200 Brazil; 4 University of North Carolina Hospitals, US 101 Manning Drive, Campus Box #7510, Chapel Hill, NC 27599 USA

**Keywords:** Uterine fibroid embolization, Superior hypogastric nerve block, Spinal anesthesia, Postprocedural pain, Opioid-sparing analgesia, Quasi-randomized study

## Abstract

**Purpose:**

To compare early postprocedural pain and rescue opioid use after uterine fibroid embolization (UFE) in patients treated with superior hypogastric nerve block (SHNB) versus spinal anesthesia.

**Materials and methods:**

This single-center prospective quasi-randomized comparative study included consecutive women with symptomatic intramural uterine fibroids treated between March 2022 and October 2023. Patients were allocated according to a predefined alternating sequence to SHNB or spinal anesthesia. Allocation concealment was not used, and patients, treating teams, and outcome assessors were not blinded. Pain was assessed at 1, 6, and 18 h after embolization using a 0–10 visual numeric scale. Rescue analgesia consisted of 3 mg intravenous morphine for moderate or severe pain according to a standardized institutional protocol. The primary endpoint was any rescue morphine use during the 18-h postprocedural observation period. Secondary endpoints were total rescue morphine dose and pain scores over time. Pain trajectory was analyzed using linear mixed-effects models, and rescue morphine outcomes were analyzed using modified Poisson and Poisson regression models.

**Results:**

Forty-two patients were included, with 21 in each group. Baseline characteristics were reasonably balanced, with all absolute standardized mean differences below 0.40. Rescue morphine use occurred in 13 of 21 patients (61.9%) in each group (risk ratio, 1.00; 95% confidence interval (CI), 0.62–1.61). No significant treatment-by-time interaction was observed for pain trajectory (*P* = .650), and time-specific between-group differences in pain scores were not significant at 1, 6, or 18 h. In adjusted analyses, treatment group was not associated with pain trajectory, any rescue morphine use, or total rescue morphine dose. Larger fibroid diameter was associated with higher pain burden and greater morphine requirement. SHNB was technically successful in all patients and was not associated with block-related complications during the assessed follow-up window.

**Conclusion:**

In this small prospective quasi-randomized comparative study, no detectable between-group differences were observed in rescue morphine use or early postprocedural pain scores between SHNB and spinal anesthesia after UFE. These exploratory findings support the feasibility of SHNB as a procedural analgesic strategy but should not be interpreted as evidence of equivalence or noninferiority.

**Graphical Abstract:**

**Figure:**
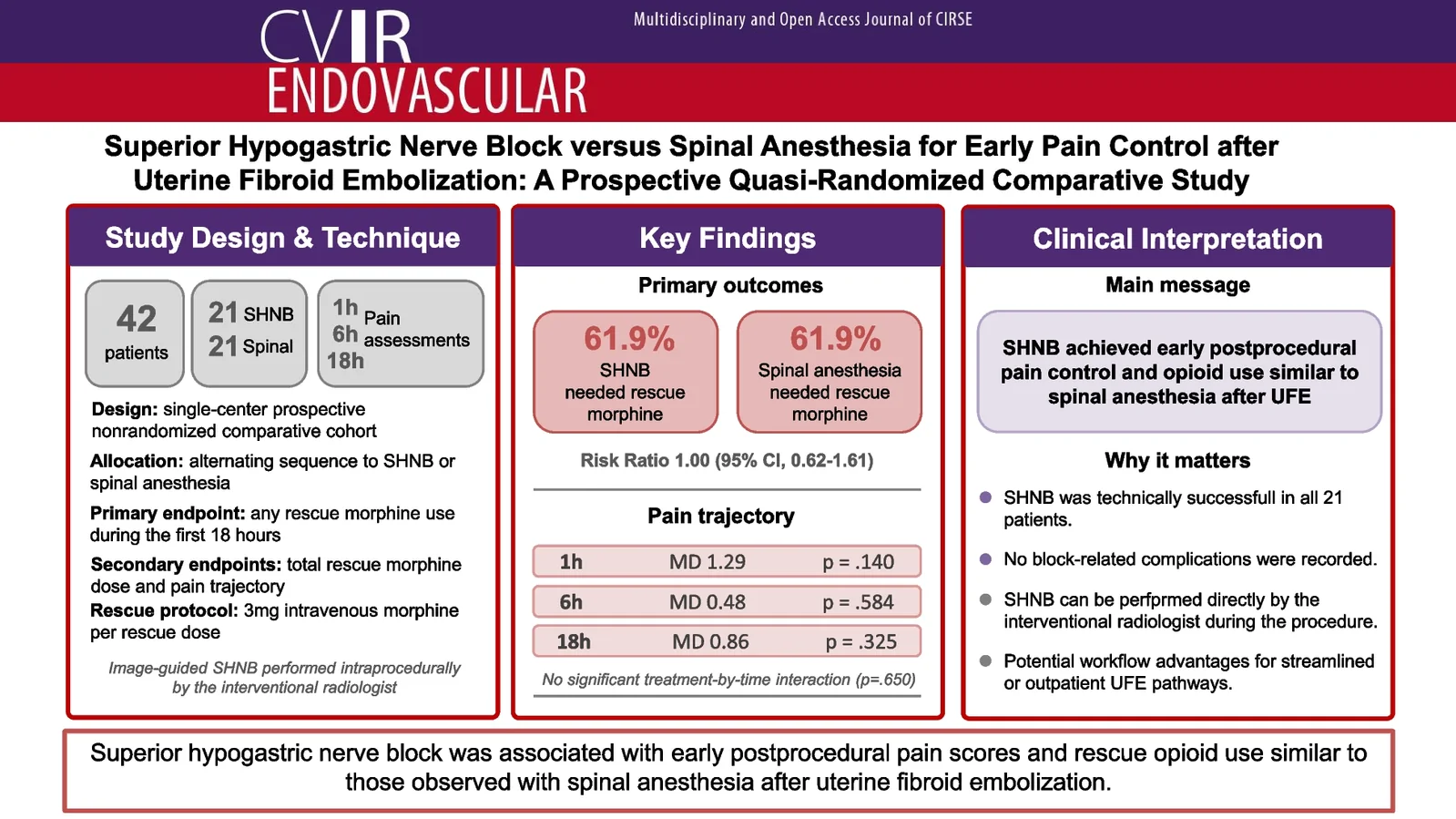

**Supplementary Information:**

The online version contains supplementary material available at 10.1186/s42155-026-00749-w.

## Introduction

Uterine fibroid embolization (UFE) is a well-established minimally invasive treatment for symptomatic uterine fibroids and an accepted alternative to surgery in selected patients. Despite its effectiveness, postprocedural pain remains a major challenge during the first 24 h after embolization and may contribute to longer observation times, greater opioid exposure, and increased health care utilization [1, 2]. Current pain management strategies frequently rely on opioid-based regimens, which may be effective but are also associated with nausea, constipation, pruritus, sedation, and the broader risks of opioid overuse [2, 3].

Spinal anesthesia has been used as an analgesic strategy in selected patients undergoing UFE because it can provide potent early pain control [4]. However, neuraxial techniques are not without drawbacks, including urinary retention requiring catheterization, prolonged immobilization, pruritus, constipation, and postspinal headache [4, 5]. These effects may be particularly relevant when simplified recovery pathways or outpatient treatment models are being considered.

Superior hypogastric nerve block (SHNB) has emerged as a targeted alternative for pelvic pain control in gynecologic and interventional radiology settings [6, 7]. In UFE, prior retrospective, case–control, and randomized studies suggest that SHNB may reduce immediate postprocedural pain, opioid consumption, and antiemetic requirements and may facilitate same-day discharge in selected settings [8–12]. However, prospective direct comparisons between SHNB and spinal anesthesia remain limited [13]. The purpose of this study was to compare early postprocedural pain scores and rescue opioid use after UFE in patients treated with SHNB versus spinal anesthesia.

## Materials and methods

### Study design and participants

This single-center prospective quasi-randomized comparative study included consecutive women undergoing UFE at a teaching hospital in Brazil between March 2022 and October 2023. The study was approved by the institutional ethics committee (CAAE 49887021.7.0000.5505), and all participants provided written informed consent before enrollment.

Eligible patients were women aged 18 years or older with symptomatic uterine fibroids referred for elective UFE, magnetic resonance imaging (MRI)-confirmed intramural fibroid disease, and ability to understand the pain scale and provide informed consent. Exclusion criteria were refusal to participate, pregnancy, suspected gynecologic malignancy, active pelvic infection, severe uncorrectable coagulopathy, contraindication to angiography or embolization, known allergy or contraindication to the study analgesic medications, contraindication to spinal anesthesia or SHNB, inability to complete postprocedural pain assessment, or incomplete procedural or follow-up documentation for the predefined 18-h observation period.

Patients were allocated according to a predefined alternating sequence to one of two analgesic strategies: SHNB or spinal anesthesia. Because allocation was based on alternation rather than computer-generated randomization, the study was designed, analyzed, and reported as a prospective quasi-randomized comparative study. Allocation concealment was not used; therefore, investigators could predict the subsequent assignment. Patients, treating physicians, nursing staff, and outcome assessors were not blinded to the analgesic strategy. No formal a priori sample-size calculation was performed; the sample represented a pragmatic prospective cohort of consecutive eligible patients treated during the study period.

### Analgesic strategies and embolization procedure

In the SHNB group, the block was performed by the interventional radiologist using an anterior percutaneous approach. The superior hypogastric plexus topography, located anterior to the L5-S1 vertebral bodies, was identified with cone-beam computed tomography and navigation guidance. After insertion of a 22-gauge needle, iodinated contrast material was injected to confirm appropriate position by the dispersion pattern, followed by administration of 10 mL of ropivacaine 10 mg/mL and 1 mL of dexamethasone 10 mg/2.5 mL in a single infusion. Dexamethasone was added according to institutional practice, with the intended rationale of prolonging the local anti-inflammatory and analgesic effect of the block; its independent contribution was not evaluated in this study. Sedation during SHNB and UFE was administered according to institutional practice and patient tolerance, without routine deep sedation or general anesthesia for the block.

In the spinal anesthesia group, spinal anesthesia was performed by the anesthesia team using a standard lumbar subarachnoid approach. After confirmation of appropriate needle position by cerebrospinal fluid return, intrathecal local anesthetic was administered in combination with 150 µg of morphine. The local anesthetic was 0.5% hyperbaric bupivacaine, with the dose individualized by the anesthesia team according to institutional practice and patient characteristics. The administered dose ranged from 10 to 15 mg (2.0 to 3.0 mL), with a median dose of 12.5 mg [10.0–15.0 mg].

All patients in the spinal anesthesia group underwent bladder catheterization as part of neuraxial management.

No routine prophylactic systemic analgesic or anti-inflammatory medication was administered uniformly to all patients before or immediately after UFE. The analgesic strategy under study therefore consisted of either SHNB or spinal anesthesia, followed by a standardized rescue protocol with intravenous morphine as needed during the postprocedural observation period. Antiemetic or other supportive medications were permitted when clinically indicated and were not considered study endpoints.

UFE was performed using the standard institutional technique with highly compressible calibrated microspheres. All procedures in this cohort were performed through common femoral arterial access. Transradial access was not used during the study period; therefore, the potential influence of radial access on postprocedural ambulation, mobility, and pain could not be evaluated. The numbers of vials used in the 300–500 μm, 500–700 μm, and 700–900 μm size ranges were recorded. Embolization endpoints included pruning of the perifibroid vascular tree.

### Postprocedural assessment and outcomes

After embolization, patients remained under hospital observation for pain monitoring and postprocedural care. Pain was assessed by the nursing team at 1, 6, and 18 h after completion of the procedure using a 0–10 visual numeric pain scale applied with verbal descriptors and a faces pain scale. Mild pain was defined as a score of 1–2, moderate pain as a score of 3–7, and severe pain as a score of 8–10.

A standardized institutional rescue analgesia protocol was used in both groups. When moderate or severe pain was reported at a scheduled assessment or at any other time during the 18-h postprocedural observation period, 3 mg of intravenous morphine was administered by the nursing team, followed by clinical reassessment according to institutional practice. The total number and timing of rescue doses were recorded. One rescue dose corresponded to 3 mg of intravenous morphine. No fixed maximum cumulative morphine dose was prespecified in the study protocol; additional doses required clinical reassessment and were withheld or delayed in the presence of excessive sedation, respiratory depression, severe nausea or vomiting, hemodynamic instability, or physician/nursing concern.

Adverse events were recorded during the intraprocedural period and throughout the 18-h postprocedural observation period before discharge, based on procedural documentation, physician assessment, and structured nursing records. Events were classified according to the Cardiovascular and Interventional Radiological Society of Europe (CIRSE) adverse event classification system using available clinical documentation.

Baseline variables included age, weight, height, body mass index, preprocedural uterine volume, number of fibroids, largest fibroid diameter, and coexisting adenomyosis on MRI. The primary endpoint was any rescue intravenous morphine use during the 18-h postprocedural follow-up period. Secondary endpoints were total rescue morphine dose and pain scores at 1, 6, and 18 h after embolization. Additional procedural outcomes included technical success of SHNB, time required to perform the block, arterial access route, microsphere use, and procedure-related adverse events within the assessed follow-up window.

### Statistical analysis

Continuous variables were summarized as mean ± standard deviation or median [interquartile range], as appropriate, and categorical variables as counts and percentages. Because treatment allocation was quasi-randomized by alternating sequence rather than truly randomized with concealment, baseline comparability was assessed primarily using absolute standardized mean differences rather than significance testing.

Any rescue morphine use was analyzed using modified Poisson regression with robust standard errors to estimate risk ratios with 95% confidence intervals. Total rescue morphine dose counts were analyzed using Poisson regression with robust standard errors and are reported as incidence rate ratios with 95% confidence intervals. Pain scores over time were analyzed using linear mixed-effects models with fixed effects for treatment group, time, and the treatment-by-time interaction and a random intercept for patient to account for repeated measurements within individuals. Time-specific between-group mean differences were obtained from this model.

Because the treatment-by-time interaction was not statistically significant, a parsimonious adjusted mixed-effects model without interaction was also fit to estimate the overall association between treatment group and pain trajectory after adjustment for largest fibroid diameter, number of fibroids, and coexisting adenomyosis. Prespecified adjusted modified Poisson and Poisson regression models for rescue morphine outcomes included the same covariates. Effect estimates and 95% confidence intervals were emphasized to support clinical interpretation rather than reliance on *P* values alone. There were no missing data for the variables included in the analysis; therefore, no imputation was performed. All tests were two-sided, and *P* < 0.05 was considered statistically significant. Statistical analyses were performed using R version 4.2.1 [14].

## Results

### Study population and baseline characteristics

A total of 42 women were included, with 21 patients allocated to SHNB and 21 allocated to spinal anesthesia. There were no missing data for the variables included in the analysis. Baseline demographic and lesion characteristics were broadly comparable between groups, with all absolute standardized mean differences below 0.40 (Table 1). Mean age was 39.1 ± 6.6 years in the SHNB group and 40.8 ± 6.8 years in the spinal anesthesia group. Mean body mass index was 28.6 ± 5.6 kg/m^2^ and 26.8 ± 3.4 kg/m^2^, respectively. Median preprocedural uterine volume was 356.0 mL [267.0–600.0] in the SHNB group and 397.0 mL [200.0–790.0] in the spinal anesthesia group. Median largest fibroid diameter was 6.4 cm [4.8–7.3] and 5.5 cm [3.5–7.3], respectively. Coexisting adenomyosis was present in 2 of 21 patients (9.5%) in the SHNB group and 5 of 21 patients (23.8%) in the spinal anesthesia group.

**Table 1:** Baseline and procedural characteristics

Characteristic	Superior hypogastric nerve block (*n* = 21)	Spinal anesthesia (*n* = 21)	Absolute SMD
Age, years	39.1 ± 6.6	40.8 ± 6.8	0.26
Weight, kg	76.2 ± 16.4	72.0 ± 11.3	0.30
Height, m	1.63 ± 0.08	1.64 ± 0.05	0.07
Body mass index, kg/m^2^	28.6 ± 5.6	26.8 ± 3.4	0.39
Uterine volume, mL	356.0 [267.0–600.0]	397.0 [200.0–790.0]	0.07
Number of fibroids	3.0 [1.0–6.0]	4.0 [2.0–6.0]	0.02
Largest fibroid diameter, cm	6.4 [4.8–7.3]	5.5 [3.5–7.3]	0.33
Coexisting adenomyosis, *n*/*N* (%)	2/21 (9.5)	5/21 (23.8)	0.38
Common femoral arterial access, *n*/*N* (%)	21/21 (100)	21/21 (100)	-
Technical success of nerve block, *n*/*N* (%)	21/21 (100)	-	-
Block time, min	12.0 [10.0–15.0]	-	-
Embolic particles 300–500 μm, vials	0.48 ± 0.98	0.62 ± 1.20	-
Embolic particles 500–700 μm, vials	2.90 ± 3.49	3.29 ± 2.51	-
Embolic particles 700–900 μm, vials	1.38 ± 2.09	0.67 ± 1.53	-

Values are presented as mean ± standard deviation, median [interquartile range], or number/total number (percentage), as appropriate. Absolute standardized mean differences (SMDs) are provided to assess baseline balance between groups in this quasi-randomized comparative cohort

### Procedural characteristics

All UFE procedures were performed through common femoral arterial access. SHNB was technically successful in all 21 patients. The time required to perform the block ranged from 6 to 22 min, with a median of 12.0 min [10.0–15.0]. No block-related complications were recorded. Microsphere use was similar between groups, with the 500–700 μm size range being the most frequently used in both treatment arms. Detailed procedural characteristics are shown in Table 1.

### Primary outcome

Rescue morphine use was identical in both groups: 13 of 21 patients (61.9%) in the SHNB group and 13 of 21 patients (61.9%) in the spinal anesthesia group required at least one rescue dose during follow-up (risk ratio, 1.00; 95% CI, 0.62–1.61; *P* = 1.000) (Table 2). The absolute risk difference was 0.0 percentage points.

**Table 2:** Primary and secondary outcomes

Outcome	Superior hypogastric nerve block (*n* = 21)	Spinal anesthesia (*n* = 21)	Effect estimate (95% CI)	*P* value
Pain score at 1 h	2 [0–7]	1 [0–4]	Mean difference, 1.29 (− 0.42 to 2.99)	.140
Pain score at 6 h	3 [1–5]	2 [1–4]	Mean difference, 0.48 (− 1.23 to 2.18)	.584
Pain score at 18 h	4 [1–6]	2 [0–5]	Mean difference, 0.86 (− 0.85 to 2.56)	.325
Any rescue morphine use, *n*/*N* (%)	13/21 (61.9)	13/21 (61.9)	Risk ratio, 1.00 (0.62 to 1.61)	1.000
Rescue morphine doses, mean ± SD	1.24 ± 1.22	1.14 ± 1.24	Incidence rate ratio, 1.08 (0.59 to 2.00)	.797
Rescue morphine doses, median [IQR]	1 [0–2]	1 [0–2]	-	-
Total rescue morphine, mg, mean ± SD	3.71 ± 3.66	3.43 ± 3.71	-	-
Total rescue morphine, mg, median [IQR]	3 [0–6]	3 [0–6]	-	-

Pain scores were assessed at 1, 6, and 18 h using a 0–10 visual numeric pain scale. Effect estimates represent between-group mean differences for pain scores and regression-based estimates for rescue morphine outcomes, with corresponding 95% confidence intervals

### Secondary outcomes

Total rescue morphine dose was similar between groups. Mean rescue dose counts were 1.24 ± 1.22 in the SHNB group and 1.14 ± 1.24 in the spinal anesthesia group, corresponding to mean total rescue morphine amounts of 3.71 ± 3.66 mg and 3.43 ± 3.71 mg, respectively. The incidence rate ratio for total rescue morphine doses was 1.08 (95% CI, 0.59–2.00; *P* = 0.797) (Table 2).

In the unadjusted mixed-effects analysis, no significant treatment-by-time interaction was observed for postprocedural pain (global* P* for interaction = 0.650). Time-specific between-group differences in pain scores were not statistically significant at any assessment time point. The estimated mean difference in pain score between SHNB and spinal anesthesia was 1.29 points (95% CI, − 0.42 to 2.99; *P* = 0.140) at 1 h, 0.48 points (95% CI, − 1.23 to 2.18; *P* = 0.584) at 6 h, and 0.86 points (95% CI, − 0.85 to 2.56; *P* = 0.325) at 18 h (Table 2).

### Adjusted analyses

In the adjusted mixed-effects model without treatment-by-time interaction, treatment group was not associated with overall pain trajectory (beta for SHNB vs spinal anesthesia, 0.62; 95% CI, − 0.73 to 1.97; *P* = 0.370) (Table 3). Larger fibroid diameter was independently associated with higher pain burden over time (beta per cm, 0.26; 95% CI, 0.02–0.51; *P* = 0.035), whereas number of fibroids and coexisting adenomyosis were not significantly associated with pain trajectory.

**Table 3:** Adjusted analyses of pain trajectory and rescue morphine outcomes

Model/covariate	Effect estimate (95% CI)	*P* value
Pain trajectory (linear mixed-effects model)		
SHNB vs spinal anesthesia	beta = 0.62 (− 0.73 to 1.97)	.370
6 h vs 1 h	beta = − 0.21 (− 1.07 to 0.64)	.625
18 h vs 1 h	beta = 0.21 (− 0.64 to 1.07)	.625
Largest fibroid diameter, per cm	beta = 0.26 (0.02 to 0.51)	.035
Number of fibroids, per fibroid	beta = 0.05 (− 0.09 to 0.19)	.480
Coexisting adenomyosis	beta = 0.04 (− 1.87 to 1.94)	.968
Any rescue morphine use (modified Poisson model)		
SHNB vs spinal anesthesia	RR = 0.95 (0.59 to 1.53)	.820
Largest fibroid diameter, per cm	RR = 1.07 (1.00 to 1.15)	.044
Number of fibroids, per fibroid	RR = 1.04 (0.99 to 1.09)	.132
Coexisting adenomyosis	RR = 1.10 (0.60 to 2.02)	.762
Total rescue morphine doses (Poisson model)		
SHNB vs spinal anesthesia	IRR = 1.06 (0.57 to 1.98)	.846
Largest fibroid diameter, per cm	IRR = 1.11 (1.03 to 1.19)	.007
Number of fibroids, per fibroid	IRR = 1.04 (0.99 to 1.09)	.135
Coexisting adenomyosis	IRR = 1.69 (0.77 to 3.71)	.189

Pain trajectory was analyzed using a linear mixed-effects model accounting for repeated measurements over time. Rescue morphine outcomes were analyzed using modified Poisson regression (any morphine use) and Poisson regression (total morphine dose). Effect estimates are reported with corresponding 95% confidence intervals

Adjusted analyses of rescue morphine outcomes showed similar findings. Treatment group was not associated with any rescue morphine use (risk ratio for SHNB vs spinal anesthesia, 0.95; 95% CI, 0.59–1.53; *P* = 0.820) or total rescue morphine dose (incidence rate ratio, 1.06; 95% CI, 0.57–1.98; *P* = 0.846). In contrast, larger fibroid diameter was associated with higher likelihood of rescue morphine use (risk ratio per cm, 1.07; 95% CI, 1.00–1.15; *P* = 0.044) and greater total rescue morphine dose (incidence rate ratio per cm, 1.11; 95% CI, 1.03–1.19; *P* = 0.007).

### Adverse events

Adverse events were assessed during the procedure and throughout the 18-h postprocedural observation period. No CIRSE grade 3 or higher adverse events occurred in either group within the assessed follow-up window. All patients in the spinal anesthesia group underwent bladder catheterization as part of planned neuraxial management; this was considered an expected component of the anesthetic pathway rather than a procedural adverse event. One patient in the spinal anesthesia group developed postanesthesia headache during the observation period, which was managed conservatively. No SHNB-related adverse events were recorded.

## Discussion

In this prospective quasi-randomized comparative study, SHNB was associated with early postprocedural pain scores and rescue opioid use that were not detectably different from those observed with spinal anesthesia after UFE. Pain trajectories at 1, 6, and 18 h were similar between groups, and the proportion of patients requiring rescue morphine was identical. Because the study was not designed or powered as an equivalence or noninferiority trial, these findings should be interpreted as exploratory and hypothesis-generating rather than as proof of equivalence between analgesic strategies.

The present study is clinically relevant because SHNB was compared with an active neuraxial analgesic strategy rather than with no block, sham treatment, or systemic analgesia alone. Prior studies have shown that SHNB may reduce immediate postprocedural pain and opioid requirements after UFE, with the most pronounced benefits often observed during the early recovery period [8–12]. Therefore, the absence of detectable differences in the present study should not be interpreted as contradicting prior evidence supporting SHNB. Rather, these data suggest that, in this institutional cohort, SHNB achieved early analgesic outcomes that were broadly similar to those observed with spinal anesthesia.

The two analgesic strategies differ substantially in workflow and resource utilization. Spinal anesthesia requires neuraxial access, anesthesia team involvement, planned bladder catheterization, and a period of neuraxial recovery. In contrast, SHNB can be performed directly by the interventional radiologist during the embolization procedure using image guidance. In this cohort, SHNB was technically successful in all patients, required a short additional procedural time, and was not associated with recorded block-related adverse events. These features may be relevant for centers seeking simplified recovery pathways or interventional radiology-driven outpatient UFE protocols.

At the same time, these results should be interpreted within the broader context of post-UFE pain, which is multifactorial and often most pronounced during the first several hours after embolization [2, 15–17]. Contemporary practice commonly uses multimodal strategies that combine regional techniques, non-opioid systemic medications, anti-inflammatory agents, antiemetics, and rescue opioids. In this framework, SHNB should be viewed as a feasible procedural component of a multimodal analgesic pathway rather than as a stand-alone replacement for comprehensive peri-procedural pain control. This point is particularly important because more than half of the patients in both groups still required rescue morphine during the 18-h observation period. This interpretation is consistent with more recent reports describing combined approaches using SHNB, intra-arterial local anesthetics, and systemic non-opioid medications in selected patients undergoing uterine fibroid embolization [18].

Procedural and patient-related factors may also influence postprocedural pain independently of the analgesic technique. In the present adjusted analyses, larger fibroid diameter was the most consistent predictor of higher pain burden and greater rescue morphine requirement. This finding is biologically plausible because greater embolized tumor volume may be associated with greater ischemic inflammatory burden. Embolic particle selection and embolization endpoint may also affect post-UFE pain. For example, initial use of larger polyvinyl alcohol particles has been associated with lower pain scores during and after UFE without compromising 6-month imaging or clinical outcomes [19].

The concern that spinal anesthesia is not universally used for UFE is valid. Many centers rely primarily on multimodal systemic analgesia, nonsteroidal anti-inflammatory medications, intra-arterial local anesthetics, regional blocks, or combinations of these approaches. The objective of this study was not to promote spinal anesthesia as a universal comparator or standard of care. Rather, spinal anesthesia was an established active analgesic pathway at the participating institution, allowing a pragmatic comparison between SHNB and a potent neuraxial strategy in an inpatient or overnight-observation model. For example, intra-arterial lidocaine administered immediately after embolization has been reported to reduce pain during the first hours after the procedure and to decrease short-term morphine requirements without clear differences in later pain scores or fibroid infarction rates at follow-up [17, 20].

This study has several strengths. Data collection was prospective, both analgesic strategies were evaluated within the same institutional setting, rescue opioid administration followed a standardized protocol, and the statistical analysis accounted for repeated pain measurements and the quasi-randomized allocation scheme. The use of effect estimates with 95% confidence intervals also provides more clinically interpretable information than *P* values alone.

Several limitations should be acknowledged. First, treatment allocation followed an alternating sequence rather than concealed randomization. Although this approach created balanced groups, it remains vulnerable to selection bias because investigators could predict the next assignment. Second, neither patients nor treating and assessing teams were blinded, which is particularly important for subjective pain outcomes. Third, the sample size was modest, no formal power calculation was performed, and the confidence intervals were relatively wide; therefore, modest benefit or harm associated with either strategy cannot be excluded. Fourth, follow-up was limited to the 18-h postprocedural observation period and did not evaluate ambulation time, discharge efficiency, same-day discharge rates, antiemetic use, patient satisfaction, or short-term healthcare reutilization. Fifth, all procedures were performed through femoral access, so the potential impact of radial access on mobility and pain could not be assessed. Finally, dexamethasone was used as part of the institutional SHNB protocol; therefore, the independent effect of ropivacaine versus dexamethasone could not be separated. Because dexamethasone may exert systemic anti-inflammatory effects, its use also represents a minor potential confounding factor when comparing SHNB directly with spinal anesthesia.

Overall, the present findings support SHNB as a feasible procedural analgesic strategy for UFE. Within the limitations of this small prospective quasi-randomized cohort, no detectable differences were observed between SHNB and spinal anesthesia in early postprocedural pain scores or rescue opioid use. Future studies should evaluate SHNB within contemporary multimodal recovery pathways, ideally using concealed randomized allocation and incorporating clinically relevant outcomes such as opioid-sparing effects, ambulation time, discharge efficiency, antiemetic use, and patient-reported recovery.

## Conclusion

In this small prospective quasi-randomized comparative study, no detectable between-group differences were observed in rescue morphine use or early postprocedural pain scores between SHNB and spinal anesthesia after UFE. These exploratory findings support the feasibility of SHNB as an interventional radiology-delivered procedural analgesic strategy, but they should not be interpreted as evidence of equivalence or noninferiority. Larger randomized comparative studies within contemporary multimodal recovery pathways are warranted.

## Supplementary Information

Supplementary Material 1: Table S1. Exploratory Univariable Associations of Baseline Characteristics with Postprocedural Pain and Rescue Morphine Outcomes.

## Data Availability

The data and materials supporting the findings of this study are available from the authors upon reasonable request.
