# Supplementary material for: Superior hypogastric nerve block versus spinal anesthesia for early pain control after uterine fibroid embolization: a prospective quasi-randomized comparative study
Source: CVIR Endovasc. 2026 Aug 27;9:107. doi: 10.1186/s42155-026-00749-w (PMC13518766; doi:10.1186/s42155-026-00749-w)
Supplement: Supplementary file 1 [file 42155_2026_749_MOESM1_ESM.docx]

**Supplementary Table S1. Exploratory Univariable Associations of Baseline Characteristics with Postprocedural Pain and Rescue Morphine Outcomes**

| **Variable** | **Exploratory pain association** | **Exploratory opioid association** | **Interpretation** |
| --- | --- | --- | --- |
| Largest fibroid diameter | SHNB: correlated with 6-h pain (Spearman rho = 0.462; P = .035). Spinal anesthesia: correlated with 1-h pain (Spearman rho = 0.465; P = .034). | In adjusted models, larger fibroid diameter was associated with any rescue morphine use (RR per cm = 1.07; 95% CI, 1.00-1.15; P = .044) and total rescue morphine dose (IRR per cm = 1.11; 95% CI, 1.03-1.19; P = .007). | Most consistent predictor of higher early pain burden and opioid requirement. |
| Number of fibroids | Spinal anesthesia: correlated with 6-h pain (Spearman rho = 0.561; P = .008). No significant correlation in SHNB. | Not significantly associated with any rescue morphine use (RR per fibroid = 1.04; 95% CI, 0.99-1.09; P = .132) or total rescue morphine dose (IRR = 1.04; 95% CI, 0.99-1.09; P = .135). | Possible exploratory signal for intermediate pain in the spinal anesthesia group, but not confirmed for opioid outcomes. |
| Age | No significant correlation with pain at 1 h, 6 h, or 18 h in either group. | Not retained as a key predictor in revised outcome models. | No clear signal. |
| Weight | No significant correlation with pain at any time point in either group. | Not retained as a key predictor in revised outcome models. | No clear signal. |
| Height | No significant correlation with pain at any time point in either group. | Not retained as a key predictor in revised outcome models. | No clear signal. |
| Uterine volume | No significant correlation with pain at any time point in either group. | Not retained as a key predictor in revised outcome models. | No clear signal. |
| Coexisting adenomyosis | Not tested in the exploratory Spearman table. | Not associated with pain trajectory (beta = 0.04; 95% CI, -1.87 to 1.94; P = .968), any rescue morphine use (RR = 1.10; 95% CI, 0.60-2.02; P = .762), or total rescue morphine dose (IRR = 1.69; 95% CI, 0.77-3.71; P = .189). | No clear signal in this cohort. |

Supplementary Table S1. Exploratory associations between baseline lesion characteristics and early postprocedural pain or rescue morphine outcomes after uterine fibroid embolization. Univariable pain associations are presented as Spearman correlation coefficients from the exploratory analysis, whereas opioid-related outcomes are summarized from the prespecified regression models in the main manuscript to highlight consistency of signal across analyses.
